# Mites present in small terrestrial mammals in a preserved unit inside the Atlantic rainforest including pathogen monitoring

**DOI:** 10.1007/s10493-026-01136-9

**Published:** 2026-04-18

**Authors:** Isabella Pereira Pesenato, Fernando de Castro Jacinavicius, Ricardo Bassini-Silva, Jaciara de Oliveira Jorge Costa, Herbert Sousa Soares, Thiago Fakelmann, Giovanna Nosberto Castelli, Mayara Bispo de Oliveira, Guiherme José da Costa Silva, Valeria Castilho Onofrio, Fernanda Aparecida Nieri-Bastos, Arlei Marcili

**Affiliations:** 1 Departamento de Biologia Animal, Instituto de Biologia, Universidade Estadual de Campinas, Campinas, SP Brazil; 2 Departamento de Medicina Veterinária Preventiva e Saúde Animal, Faculdade de Medicina Veterinária e Zootecnia, Universidade de São Paulo, São Paulo, SP Brazil; 3 Laboratório de Coleções Zoológicas, Instituto Butantan, São Paulo, SP Brazil; 4 Universidade Santo Amaro, São Paulo, SP Brazil; 5 Faculdade Anclivepa, Medicina Veterinária, São Paulo, SP Brazil

**Keywords:** Ectoparasite, Rodent, Marsupial, *Rickettsia*, DNA

## Abstract

Ectoparasitic mites have been studied for many years in the medical and veterinary fields, particularly for their association with humans and domesticated animals. At environments with little anthropic actions is possible to learn more about their interaction with natural or primary hosts. In this study, ectoparasitic mites of rodents and mammals of the Legado das Águas reserve were studied. The reserve is located inside the Brazilian Atlantic Rainforest with high levels of conservation due to being mainly used for ecotourism. The objective of this study was to explore ectoparasitic mites in three areas of the reserve, and search rickettsiae bacteria in some sampled specimens. Mites were collected from hosts using Sherman and Tomahawk traps and later identified using taxonomic keys. A total of 6,947 mites were collected belonging to three families (Laelapidae, Macronyssidae and Trombiculidae) on 478 hosts. Individually selected mite samples (*N* = 186) were subjected to DNA extraction and further tested for occurrence of Rickettsia, however, no sample retrieved positive results. This study reports new mite records for hosts and localities and explores the dynamics of the ecology of mites and their hosts in a particular area inside the Atlantic Rainforest.

## Introduction

Ectoparasites are commonly found in natural environments, living along with small terrestrial mammals, such as rodents and marsupials, causing little or no harm, being the result of many years of coevolution (Cançado et al. 2017). These parasites are part of a prominent role concerning zoonotic diseases, acting as vectors to humans or other groups of animals. Among them, ticks are the most studied regarding the presence of *Rickettsia* bacteria, leaving other group of mites in a neglected landscape (Souza et al. 2021).

Mites are chelicerate arthropods with a wide variety of feeding habits, and some families can present parasitic habits, primarily interacting with small mammals (Walter and Proctor 2013). Association patterns of these ectoparasites with specific hosts have been studied in the Neotropical region, with differences between degraded and preserved areas (Jacinavicius et al. 2018a; Bassini-Silva et al. 2020).

The most common group of mites found parasitizing rodents and marsupials are mesostigmatids (Mesostigmata), followed by chiggers (Trombidiformes: Trombiculidae *sensu lato*) with already described roles in some zoonotic diseases such as rickettsialpox (caused by *Rickettsia akari* vectored by mesostigmatids) and scrub typhus (caused by *Orientia tsutsugamushi* transmitted by chiggers) (Herrera-Mares et al. 2022). Within the Mesostigmata two families have high relevancy specially when it comes to small mammals, the family Macronyssidae includes specific and generalist species with hematophagous habits (Bassini-Silva et al. 2020) and the family Laelapidae, whose species are mainly associated with rodents, found on their body surface or nests, but not demonstrating hematophagous habits (Mašán and Fenda 2010). Although, some studies are being conducted to investigate pathogens in these two groups of mites (Reeves et al. 2007; Jacinavicius et al. 2019; Bassini-Silva et al. 2023), the number is minimal when compared to other ectoparasites, such as ticks.

The Brazilian Atlantic Rainforest is one of the 35 biodiversity hotspots on the planet, sheltering regions with high levels of endemism but heavily threatened by human activities, replaced by pastures and monocultures, resulting in a loss of habitat for animals and increasing human-animal interaction with parasites exchange (Linhares de Rezende et al. 2015; Vidal-Martínez and Wunderlich 2017). Most of what remains of the Atlantic Forest is under the protection of laws and forest reserves, which encourage the maintenance of these areas with the introduction of scientific research, partnerships with conservationists and tourism to sustain local biodiversity (Mittermeier et al. 2004; Legado das Águas 2018).

The objective of this study was to identify the species of mites that were collected from rodents and marsupials in three areas of the Atlantic Rainforest reserve Legado das Águas - Reserva Votorantim, São Paulo State, Brazil, and detection of bacterial species of the genus *Rickettsia* in these mites.

## Materials and methods

### Specimens collection

**Fig. 1:**
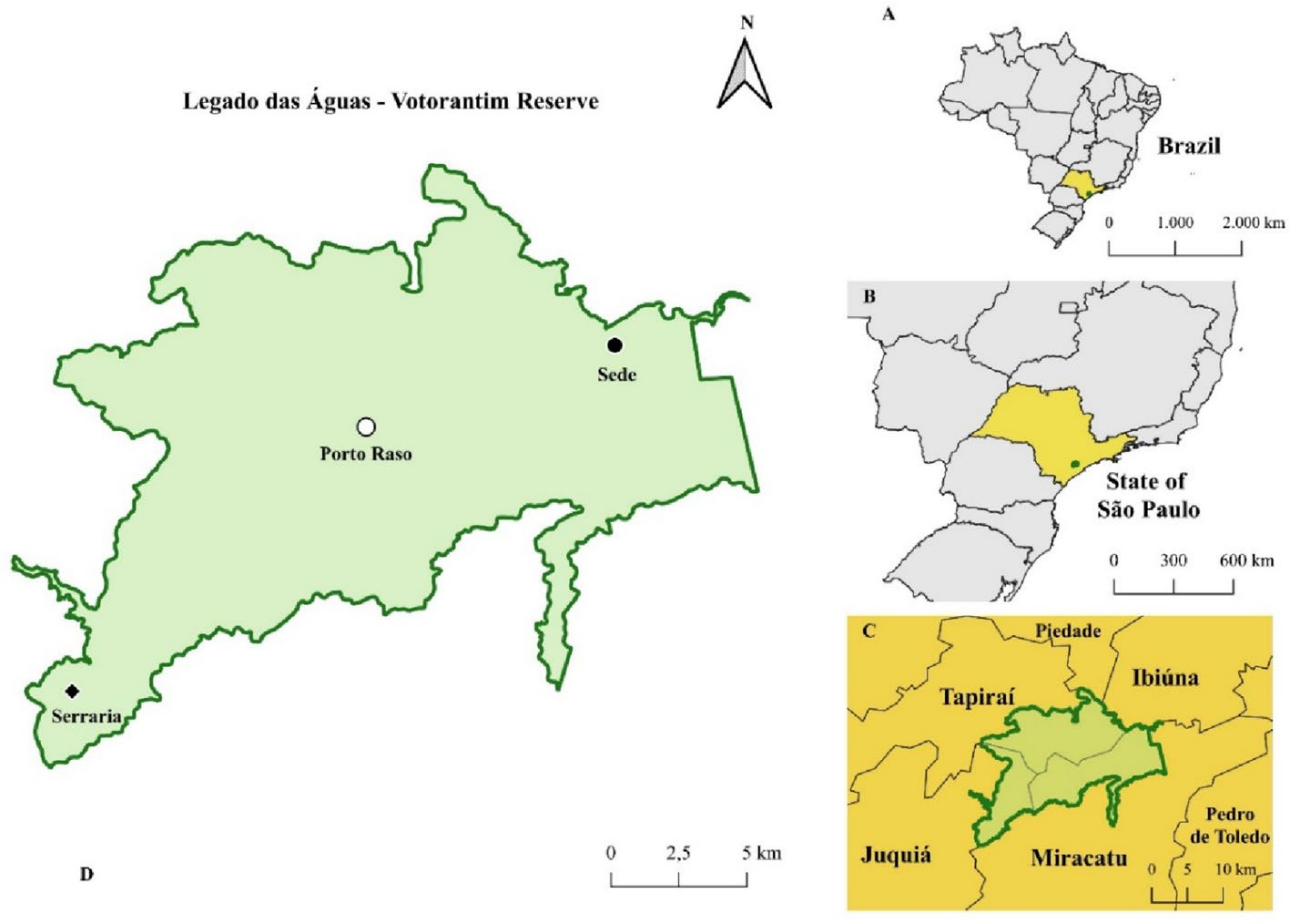
Map showing the collection area. (**A**) Brazil and State of São Paulo highlighted; (**B**) Position of the reserve inside São Paulo’s state; (**C**) municipalities surrounding the reserve area; (**D**) Collection sites within the Legado das Águas limit (QGIS, 2024)

The mites were collected in the private reserve Legado das Águas - Reserva Votorantim, Miracatu, São Paulo, Brazil, located in a fragment of Atlantic Forest, with approximately 75% of the total area composed of dense primary ombrophilous forest. Within the perimeter of the reserve, three areas were chosen for capture: Sede (24° 1’ 49.51” S, 47° 21’ 8.36” W), Porto Raso (24° 3’ 25.90” S, 47° 26’ 30.07” W) and Serraria (24° 9’ 9.63” S, 47° 32’ 53.49” W), the locality and the areas are better shown in Fig. 1.

Between January 2018 and December 2021, eight field collection campaigns were conducted, each lasting on average 7–12 days. Three campaigns were carried out in the Sede area (January, July, and December 2018; 6 days each), three in Porto Raso (July 2019, February 2020, and October 2021; 6 days each), and two longer campaigns in Serraria (September and December 2022; 9 days each). Due to interruptions caused by the COVID-19 pandemic, the sampling effort was standardized to a total of 18 field days in each locality.

The trails chosen were based on the vegetation and tracks of wild animals. In total, 240 traps (120 Sherman and 120 Tomahawk) were used in each campaign, and the small mammals were captured using bait made with a mixture of sardines, cornmeal, coconut oil, vanilla, and peanut paste. After capture, the animals were anesthetized with ketamine hydrochloride (15-30 mg/kg), and following sample collection and recovery, the rodents and marsupials were identified using taxonomic keys (Bonvicino et al. 2008; Faria 2019) then returned to the wild at the same site as captured. All mites were collected using tweezers and stored in a microtube containing absolute ethanol.

### Mite morphological identification

After collecting, the samples were sent to the Laboratory of Zoological Collections of the Instituto Butantan (LCZ-IB) for taxonomic determination.

The mites were slide-mounted using Hoyer’s medium following the protocols described by Krantz and Walter (2009). For the identification of genera, we used Radovsky (2010), Brennan and Goff (1977), and Fonseca (1936). We compared our specimens at the species level with the original descriptions of all species assigned to the identified genera. The mites were counted to provide prevalence and abundance.

Images and measurements were taken using a Leica DM4000B microscope and compiled with Leica Application Suite version 2.5.0. All slide-mounted specimens were deposited in the Acarological Collection at the LCZ-IB under the accession numbers IBSP18723 - IBSP18862.

A presence/absence matrix of mites per host species was used to estimate species richness. Species accumulation curves were generated using the *specaccum* function from the vegan R package (Oksanen 2022; R Core Team, 2024). Chao1 and Bootstrap estimators were calculated to assess sampling completeness and provide lower and upper bounds of species richness (Chao 1984; Colwell and Coddington 1994).

### Molecular analysis

Part of the collected mites were stored for molecular analysis. For this, each mite was individualized in a microtube and submitted to DNA extraction using the commercial DNeasy Blood and Tissue Kit (Qiagen^®^), protocol suggested by the manufacturer was followed. After DNA extraction, each exoskeleton was recovered from the columns and slide-mounted for identification following the steps previously described.

From the extracted DNA, conventional PCRs were performed using primers that amplify endogenous genes of the mite, the small subunit (SSU) 18 S rRNA gene following the thermal cycler conditions proposed by Otto and Wilson (2001), the 16 S mitochondrial rRNA gene, following the thermal cycler conditions proposed by Mangold et al. (1998) and the Cytochrome c oxidase subunit 1 (*cox1*) gene following the thermal cycler conditions proposed by Folmer et al. (1994). All reactions included positive (DNA extracted from *Blankartia sinammaryi* [Floch and Fauran, 1956]) and negative (ultrapure water type I) controls.

Positive Samples for the endogenous genes were considered viable, thus screened for bacteria of the genus *Rickettsia*. First, a real-time polymerase chain reaction (qPCR) was performed using the primers CS-5 and CS-6 and including an internal probe to amplify a 147 bp fragment of the *gltA* gene, present in all bacteria of this genus. This reaction was performed following the protocols described by Labruna et al. (2004) and Guedes et al. (2005). All reactions included positive (DNA extracted from cell culture infected with *Rickettsia vini*) and negative (ultrapure water type I) controls. All the primers used in the molecular tests are described in the Table 1.

**Table 1:** List of primers employed to amplify partial gene fragments from mite samples and associated *Rickettsia* bacteria

Organism	Gene	Sequence 5’3’	Size (pb)	References
*Rickettsia*	*gltA* (qPCR)	CS-5: GAGAGAAAATTATATCCAAATGTTGAT	147	Guedes et al. (2005) and Labruna et al. (2004)
		CS-6: AGGGTCTTCGTGCATTTCTT		
		6-FAM d(CATTGTCGGATCCAGCCTACGGT) BHQ-1		
*Rickettsia*	*gltA*	CS-239: GCTCTTCTCATCCTATGGCTATTAT	834	Labruna et al. (2004)
		CS-1069: CAGGGTCTTCGTGCATTTCTT		
*Rickettsia*	*ompA*	Rr190.70: ATGGCGAATATTTCTCCAAAA	632	Regnery et al. (1991) and Roux et al. (1997)
		Rr190.701: GTTCCGTTAATGGCAGCATCT		
Mite	18 S rRNA	18 S–1 F: ATATTGGAGGGCAAGTCTGG	500	Otto and Wilson (2001)
		18 S-1R: TGGCATCGTTTATGGTTAG		
Mite	16 S rRNA	16 S + 1: CCGGTCTGAACTCAGATCAAGT	460	Mangold et al. (1998)
		16 S-1: GCTCAATGATTTTTTAAATTGCTGT		
Mite	*cox1*	LCO1490: GGTCAACAAATCATAAAGATATTGG	710	Folmer et al. 1994
		HCO2198: TAAACTTCAGGGTGACCAAAAAATCA		

The reactions that generated amplicons were purified with ExoSAP-IT (USB Corporation^®^, OH), using the manufacturer’s instructions, and subsequently sequenced through Sanger sequencing performed at the “Human Genome and Stem Cell Research Center of the Institute of Biosciences of the USP”. The sequences obtained were edited using the program SeqMan (Lasergene, DNAstar, Madison, Wis.) and then submitted to analysis using the program BLASTn to infer similarity with other homologous sequences already deposited in the GenBank (Altschul et al. 1990).

### Ethical statement

The Ethics Committee of the Faculty of Veterinary Medicine and Zootechny of the University of São Paulo (FMVZ-USP) under the number 6,509,131,119 approved the present study.

## Results

### Host-associations

During the sampling years, 478 mammals were collected, being 368 rodents and 110 marsupials. Of these, 283 rodents, belonging to the families Cricetidae (12 genera) and Sciuridae (1 genus), and 42 marsupials, belonging to the family Didelphidae (6 genera) were infested with mites from three families: Trombiculidae, Macronyssidae, and Laelapidae, resulting in a total of 6,988 specimens. A summary of the mite and host species identified, along with the corresponding number of specimens collected, is provided in Tables 1 and 2.

**Table 2:** Mite species collected on rodents in a preserved ecological area (Legado das Águas – Reserva Votorantim) inside the Atlantic Rainforest Biome, during 2018 to 2021

Mite species	Hosts (infested/captured)													
	*Akodon* sp. (*n* = 2) ^SR^	*Brucepattersonius* sp. (*n* = 5) ^PR^	*Delomys* sp. (*n* = 1) ^SE^	*Euryoryzomys russatus* (*n* = 225) ^SE, PR, SR^	*Guerlinguetus* sp. (*n* = 1) ^SR^	*Holochilus* sp. (*n* = 58) ^PR^	*Hylaeamys* sp. (*n* = 8) ^SE, PR^	*Nectomys squamipes* (*n* = 15) ^PR^	*Oligoryzomys* sp. (*n* = 15) ^*SE, PR, SR*^	*Oxymycterus* sp. (*n* = 26) ^*SE, PR, SR*^	*Rhipidomys* sp. (*n* = 2) ^PR^	*Sooretamys* sp. (*n* = 2) ^SE^	*Thaptomys nigrita* (*n* = 3) ^SR^	Total
	Prevalence (infested hosts/total number of hosts) Abundance													
**Family Laelapidae**														
*Androlaelaps fahrenholzi*	50 (1/2) 9	20 (1/5) 1	100 (1/1) 1	38.67 (87/225) 472	-	20.69 (12/58) 79	25 (2/8) 12	33.33 (5/15) 31	40 (6/15) 17	15.38 (4/26) 18	100 (2/2) 147	-	33.33 (1/3) 1	788
*Androlaelaps rotundus*	50 (1/2) 29	-	-	-	-	-	-	-	-	-	-	-	-	29
*Gigantolaelaps gilmorei*	-	-	100 (1/1) 1	52 (117/225) 573	-	-	25 (2/8) 6	6.67 (1/15) 4	40 (6/15) 20	-	100 (2/2) 22	50 (1/2) 5	-	631
*Gigantolaelaps oudemansi*	-	-	100 (1/1) 1	83.56 (188/225) 3027	-	3.45 (2/58) 10	75 (6/8) 77	6.67 (1/15) 17	60 (9/15) 138	-	100 (2/2) 69	50 (1/2) 20	-	3359
*Gigantolaelaps wolffsohni*	-	-	-	1.78 (4/225) 30	-	44.83 (26/58) 210	12.50 (1/8) 2	60 (9/15) 92	20 (3/15) 32	-	50 (1/2) 29	-	-	395
*Laelaps castroi*	-	-	-	9.78 (22/225) 52	-	-	12.50 (1/8) 1	-	6.67 (1/15) 4	-	50 (1/2) 2	50 (1/2) 9	-	68
*Laelaps differens*	-	-	-	9.33 (21/225) 80	-	-	-	-	13.33 (2/15) 6	3.85 (1/26) 1	50 (1/2) 3	-	-	90
*Laelaps manguinhosi*	-	-	-	-	-	12.07 (7/58) 49	-	13.33 (2/15) 29	-	-	-	-	-	78
*Laelaps paulistanensis*	-	-	-	-	-	-	-	-	6.67 (1/15) 2	-	-	-	-	2
*Laelaps thori*	-	20 (1/5) 2	-	0.44 (1/225) 1	-	1.72 (1/58) 3	-	-	-	19.23 (5/26) 97	-	-	-	103
*Laelaps* sp.	-	-	-	1.78 (4/225) 15	-	3.45 (2/58) 5	-	-	-	15.38 (4/26) 6	-	-	-	26
**Family Macronyssidae**														
*Ornithonyssus* sp.	50 (1/2) 2	60 (3/5) 36	-	-	-	3.45 (2/58) 4	-	6.67 (1/15) 1	-	80.77 (21/26) 278	-	-	-	321
**Family Trombiculidae**														
*Eutrombicola tinami*	-	-	-	0.44 (1/225) 4	100 (1/1) 128	-	-	-	-	-	-	-	-	132
*Quadraseta brasiliensis*	-	-	100 (1/1) 8	8.44 (19/225) 119	-	-	-	-	13.33 (2/15) 13	-	50 (1/2) 1	-	-	141
*Quadraseta pazca*	-	-	100 (1/1) 12	6.22 (14/225) 234	-	-	-	-	13.33 (2/15) 18	-	-	-	33.33 (1/3) 2	266
Total	40	39	23	4607	128	360	98	174	250	400	273	34	3	6429

*SE: Sede; PR: Porto Raso; SR: Serraria

The mites belonging to the order Mesostigmata correspond to two families Laelapidae and Macronyssidae. The family Laelapidae had the higher species richness in our study (four genera and eleven species), collected and associated with rodents and marsupials in the study area. Also, there were several life stages: protonymphs, deutonymphs, males, and females. Twenty-nine batches were collected in marsupials and 285 in rodents, ranging from 1 to 105 specimens each.

The species in this family belong to four different genera and were associated with both rodents and marsupials. *Androlaelaps fahrenholzi* (Berlese, 1911) was found associated with eleven and three rodent and marsupial species, respectively. In contrast,, *Androlaelaps ilhacardosoi* Gettinger and Martins-Hatano (2003) was found in associantion with marsupial, *Monodelphis* sp., while *Androlaelaps rotundus* (Fonseca, 1935) was only collected from the rodent *Akodon* sp.

**Fig. 2:**
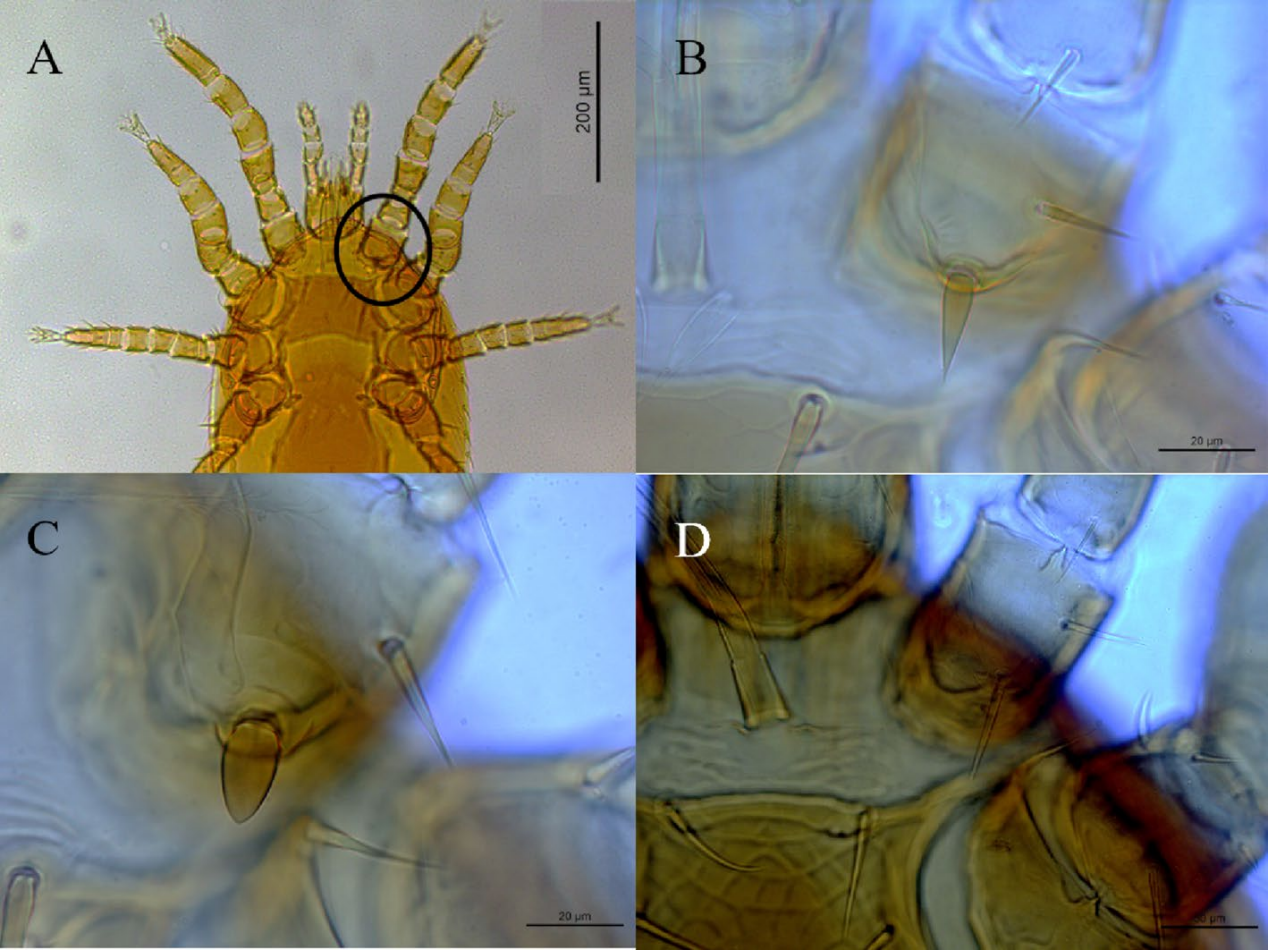
Differences observed between the spine-like setae present on the coxa I among laelapids. **A** Spine-like setae on coxa I; **B** spine of *Laelaps differens*; **C** spine of *Laelaps castroi*; **D** spine of *Laelaps thori*

The genus *Gigantolaelaps* Fonseca, 1939 was represented by three species: *Gigantolaelaps gilmorei* Fonseca, 1939, *Gigantolaelaps oudemansi* Fonseca, 1939, and *Gigantolaelaps wolffsohni* (Oudemans, 1910). The first two species were recorded parasitizing rodents and marsupials, whereas the latter has been found exclusively on rodents. In addition. five species were found associated with rodents and identified as belonging to the genus *Laelaps* Koch, 1836, as follows: *Laelaps castroi* Fonseca, 1957, *Laelaps differens* Fonseca, 1935, *Laelaps manguinhosi* Fonseca, 1935, *Laelaps paulistanensis* Fonseca, 1935, and *Laelaps thori* Fonseca, 1939 (Fig. 2). Some immature stages specimens were identified to the genus level of *Laelaps*.

Conversely, the Macronyssidae family showed the lowest richness, with species associated only with rodents. Thirty lots were collected from rodents ranging from 1 to 102 specimens per lot. Only the genus *Ornithonyssus* Sambon, 1928 was determined including species associated with four genera of rodents, *Brucepattersonius* Hershkovitz, 1998, *Nectomys* Peters, 1861, *Oxymycterus* Waterhouse, 1837 and *Holochilus* Brandt, 1835. The information on mites collected on rodents is compiled in Table 2 and the mites collected on marsupials is on Table 3.

**Table 3:** Mite species collected on marsupials in a preserved ecological area (Legado das Águas – Reserva Votorantim) inside the Atlantic Rainforest Biome, during 2018 to 2021

Mite species	Hosts (infested/captured)						
	*Didelphis aurita* (*n* = 28) ^*SE, PR, SR*^	*Gracilinanus sp.* (*n* = 1) ^SE^	*Marmosa (Micoureus) demerarae* (*n* = 3) ^SE^	*Marmosops sp.* (*n* = 2) ^SE^	*Metachirus myosurus* (*n* = 72) ^SE, PR^	*Monodelphis sp.* (*n* = 1) ^SR^	Total
	Prevalence (infested hosts/total number of hosts) Abundance						
**Family Laelapidae**							
*Androlaelaps fahrenholzi*	3.57 (1/28) 11	-	-	100 (2/2) 132	47.22 (34/72) 314	-	457
*A. ilhacardosoi*	-	-	-	-	-	100 (1/1) 1	1
*Gigantolaelaps gilmorei*	3.57 (1/28) 7	-	-	-	-	-	7
*G. oudemansi*	-	100 (1/1) 6	-	-	1.39 (1/72) 30	-	36
*Laelaps* sp.	-	-	33.33 (1/3) 3	-	1.39 (1/72) 1	-	4
**Family Trombiculidae**							
*Colicus spinosus*	-	-	-	-	-	100 (1/1) 2	2
*Eutrombicola tinami*	10.71 (3/28) 11	-	-	-	-	-	11
Total	29	6	3	132	345	3	518

*SE: Sede; PR: Porto Raso; SR: Serraria

**Fig. 3:**
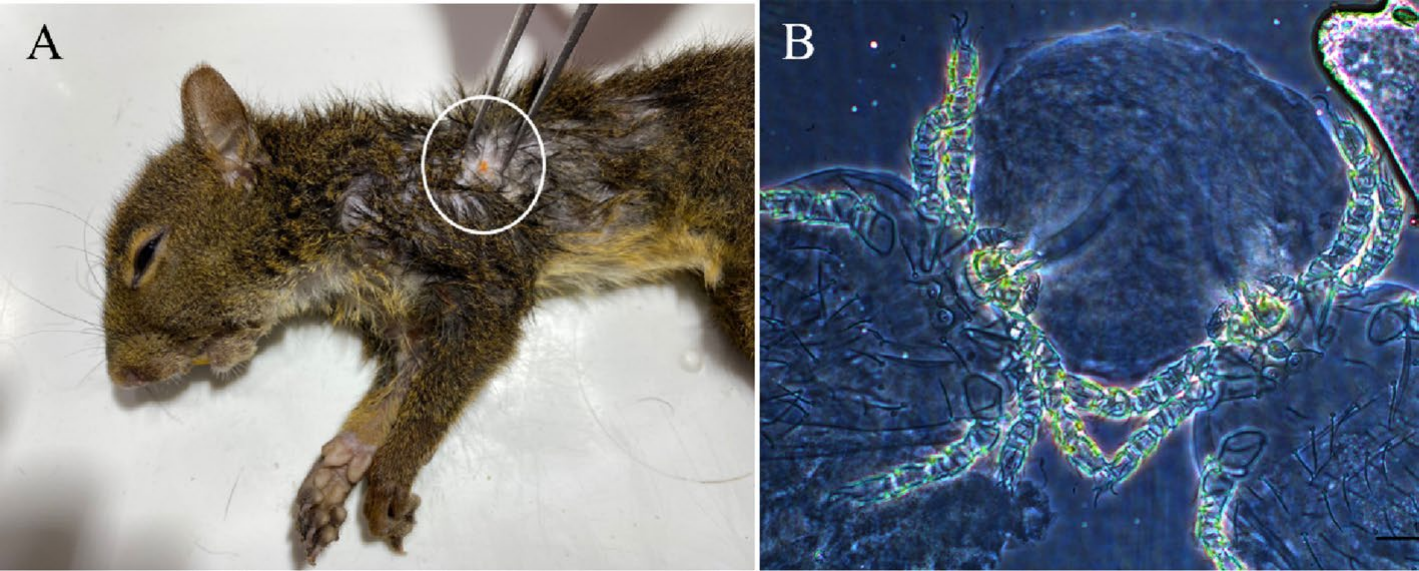
(**A**) parasitism of chigger mites over the body of *Guerlinguetus brasiliensis*. (**B**) Co-parasitism between *Quadraseta pazca* and *Quadraseta brasiliensis* attached to the same skin fragment collected from a *Euryoryzomys russatus*

The family Trombiculidae, was represented by three genera and four species. The genus *Quadraseta* Brennan, 1970 was the most abundant in this study, represented by two species, *Quadraseta pazca* (Brennan & Jones, 1964) and *Quadraseta brasiliensis* Goff & Gettinger, 1989, collected from rodents belonging to five genera. The species *Eutrombicula tinami* Oudemans, 1910 was collected from two species of rodents and one marsupial. Lastly, *Colicus spinosus* Goff & Gettinger, 1989 parasitized only the *Monodelphis* sp marsupial. Six different rodent species from the families Cricetidae and Sciuridae and two species of marsupials were found parasitized with 34 batches of mites, ranging from 1 to 50 specimens per host specimen collected. The ectoparasites were collected, parasitizing mainly the ear canal of these vertebrates, and some cases, were found on the animal’s body (Fig. 3a). Co-parasitism among species was also observed, including two specimens attached to the same skin fragment, *Q. pazca* and *Q. brasiliensis* collected from *Euryoryzomys russatus* (Wagner, 1848) (Fig. 3b).

**Fig. 4:**
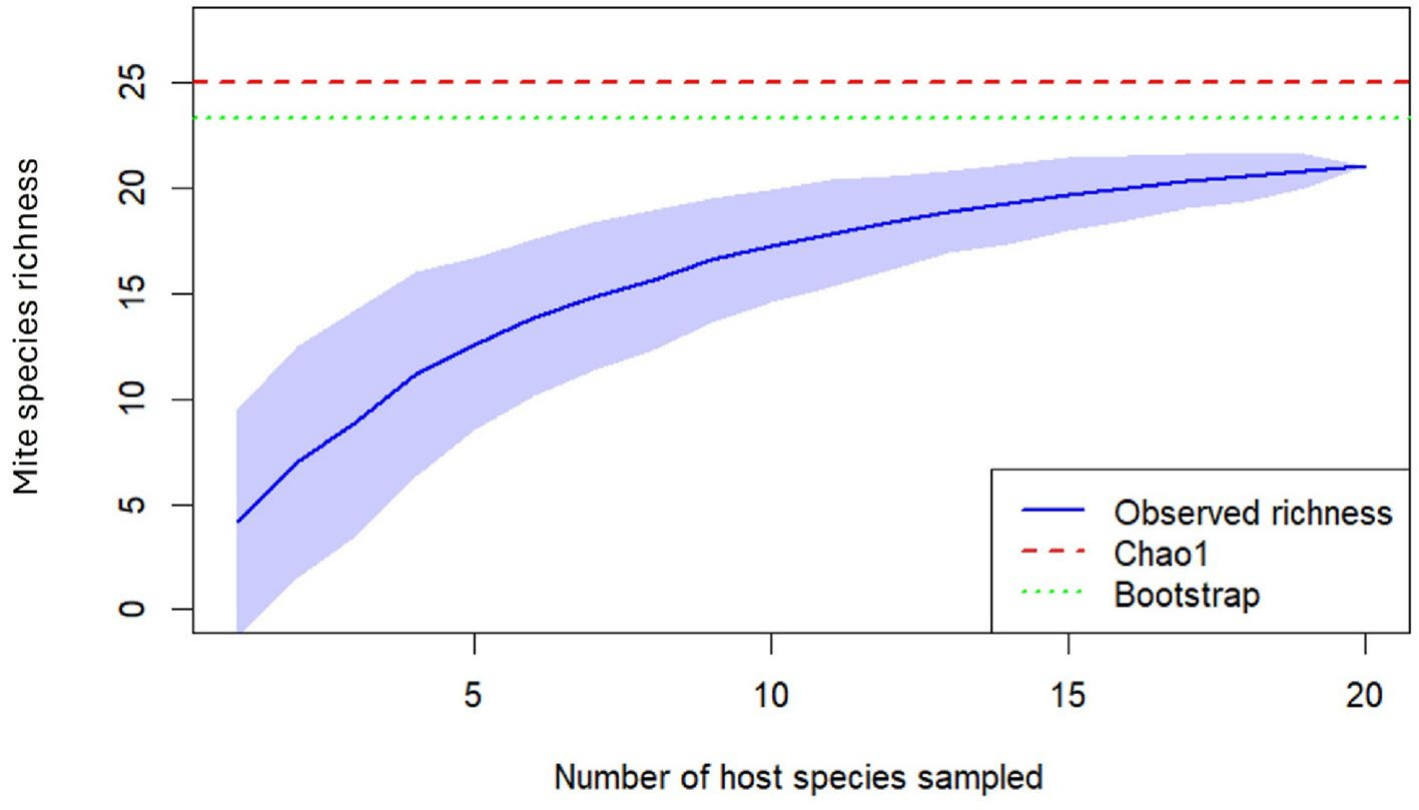
Species accumulation curve of mites by host species. The blue line represents the observed tick species richness across sampled host species, while the red dashed line shows the Chao1 richness estimator, and the green dotted line indicates the Bootstrap richness estimator. Shaded areas around the observed curve represent confidence intervals

Based on the ectoparasite species obtained and the number of hosts analyzed, the species accumulation curve has a tendency towards an asymptote (Fig. 4).

### Molecular analysis

One hundred fifteen specimens of chigger mites were processed individually for the 18 S rRNA gene, and 99 samples generated expected amplicons with the expected size, these 14 samples generated viable sequences for the species *Q. pazca* (OQ026942 to OQ0269550). The other species of chiggers did not generate viable sequences. All the 99 successful samples for the endogenous control were tested for the *gltA* gene present in all *Rickettsia* bacteria, but none showed positivity. Attempts to amplify fragments of the *cox1* gene were unsuccessful in chigger mites.

From the macronyssid mites collected, 71 were processed individually for the 16 S rRNA and the 18 S rRNA genes; of these 55 samples amplified expected amplicons for both genes and were subsequently tested for the *gltA* gene. However, none showed positivity for the genus *Rickettsia*.

## Discussion

The study showed the presence of a vast acarofauna in a preserved fragment inside the Atlantic Rainforest, interacting with rodents and marsupials in three areas of the private reserve Legado das Águas – Reserva Votorantim. In addition, there was no evidence of bacteria of the genus *Rickettsia* circulating in this group of ectoparasites that can also interact with humans.

The number of chigger species found was relatively low when compared to other studies conducted in preserved areas, as will be discussed below. Here, we identified three genera and four species in four years of collection, with no new species found. Jacinavicius et al. (2020) studied a conserved unit in the Pará state (Brazil) within the Caatinga biome and described four new species of chigger mites. In another study conducted with chigger mites collected in the São Paulo state, among 317 specimens examined, Jacinavicius et al. (2019) identified six genera and twelve species also retrieved from small terrestrial mammals (rodents and marsupials) sampled in the same biome from this study.

Here, we report a new locality record for the species *Colicus spinosus*. The previous records were in the checklist for chigger mites from Brazil (Jacinavicius et al. 2018a), which stated that this species was recorded in Brasília (Federal District) and Itapevi (São Paulo) associated with Didelphimorphia. Our record was collected in the same host genus reported from Itapevi, *Monodelphis*.

The species *Eutrombicula tinami* has been described as a neglected ectoparasite already found parasitizing humans (Bassini-Silva et al. 2019) in the same biome as collected here, with reports of trombiculiasis symptoms, such as pruritus, dermatitis, and inflammatory reactions (Pesenato et al. 2024). The researchers that conducted the field experiment had similar symptoms during the collections, but these mites have reduced sizes, making it hard to find. This mite has no preferential host association, acting as a generalist mite, and has been described as parasitizing several bird species, rodents, humans (Bassini-Silva et al. 2019), marsupials (Jacinavicius et al. 2018a), and even domestic animals that have free accession to forested areas (Cousandier et al. 2021). Here, we report the first time this mite is described as parasitizing a rodent of the genus *Guerlinguetus*.

The Neotropical genus *Quadraseta* presents a wide variety of species and is mainly associated with small terrestrial mammals (Jacinavicius et al. 2018a) and has been associated with the presence of bacteria of the genus *Orientia* in specimens collected from rodents in Chile (Silva de la Fuente et al. 2023). Jacinavicius et al. (2018b) contributed with the knowledge of *Q. brasiliensis*, reporting new hosts and localities in Brazil, and Arbex et al. (2021) reported this species in Suriname in an echimyid rodent. Here, we report a new locality for this species in the Miracatu and Tapiraí municipalities (São Paulo) and also a new host interaction with *Rhipidomys* sp.

The co-parasitism among chiggers has been highlighted by many authors (Bassini-Silva et al. 2021; Jacinavicius et al. 2021; Stekolnikov et al. 2022) since this group of mites is not very selective in terms of hosts. Jacinavicius et al. (2023) reported co-infestation of three different species of trombiculids in the same cluster collected from a cat. Also, Jacinavicius et al. (2021) reported the same occurrence in different sites along the host body. Here, we report a case of co-parasitism of *Q. pazca* and *Q. brasiliensis* collected from an *E. russatus*, inside the ear canal of this host.

Another important observed feature is the attachment placed on the host’s body. A particular preference is observed because the genus *Quadraseta* was only found inside the ear canal of the hosts. The same pattern was observed by Jacinavicius et al. (2021). Meanwhile, species of the genera *Eutrombicula* and *Colicus* have been found in the torso and abdomen of the hosts.

The mites of the family Laelapidae are often associated with rodents and, more commonly, the females belonging to this family; the other stages can be found in the nest of these hosts feeding on organic matter (Netušil et al. 2013). In the present study, we collected in the hosts, besides females, males, deutonymphs, and protonymphs. The genus *Gigantolaelaps* comprises ten species in Brazil (Furman 1972; Gettinger et al. 2011; Barros-Battesti et al. 2025), and their main character is their large size. The most collected species was *G. oudemansi*, found in rodents and marsupials, but most of the species was collected from the rodent species *E. russatus*. Our observation corroborates what was stated by Gettinger (1987); due to, he observed this same species mainly associated with the oryzomyine group (Cricetidae: Sigmodontinae). A strong association was also observed between this mite species and *Gigantolaelaps gilmorei*; meanwhile, the species *Gigantolaelaps wolffsohni* was not associated with other mites.

The species group complex *Androlaelaps rotundus* was only found interacting with rodents of the genus *Akodon*. In Paraguay, Sánchez-Martínez and Owen (2021) observed that this rodent rathers a more preserved environment. However, we only collected this host in our most degraded area (Serraria), so the number of mite specimens belonging to this species complex was reduced. Lareschi (2020) proposed that this species-group complex probably constitutes a new genus, but since it remains a species-group, those specimens were identified as that. The same situation occurs with *A. fahrenholzi*, since it has a morphometric difference between the localities of the collection (Silva de la Fuente et al. 2020). The species *Androlaelaps ilhacardosoi* was reported only once at Ilha do Cardoso – SP by Gettinger and Martins-Hatano (2003), found in a marsupial of the genus *Monodelphis*. Here, we report this species collected from the same host genus but in a different locality, Vale do Ribeira region.

Regarding the genus *Laelaps*, we report five species mainly related to rodents and rarely to marsupials. Gettinger (1992) observed that some species of this genus are host specific such as *L. castroi*, *L. differens*, *L. manguinhosi*, and *L. thori*. However, for the *L. castroi* species, we observed a different pattern, we collected this mite in five different rodent species: *E. russatus*, *Oligoryzomys* sp., *Hylaeamys* sp., *Sooretamys* sp., and *Rhipidomys* sp, as opposed to Nieri-Bastos et al. (2004) that collected this species from only one host (*Oligoryzomys* sp.). The same happened with *L. differens* and *L*. *thori*, collected from four different rodent genera. *L. manguinhosi* was only retrieved from *Nectomys squamipes* (Brants, 1827) and *Holochilus* sp., which can be explained because both rodents shared the same collection spots and agreed with Lareschi et al. (2006) results. These different patterns can be explained by the different biomes collected once Gettinger (1992) researched Brazil’s Cerrado, and this study was conducted in the Atlantic Rainforest biome with a wide variety of hosts inhabiting the same spot. The only species we observed as a host specificity was *L. paulistanensis*, associated only with rodents from the genus *Oligoryzomys*, this same pattern was observed by Lareschi et al. (2006) in Uruguay and Savchenko et al. (2021) in Argentina, but not by Barros et al. (1993) who retrieved this mite from two rodent species: *Akodon montensis* Thomas, 1913, and *Oligoryzomys nigripes* (Olfers, 1818).

In this study, we report the collection of three different genera of laelapid mites comprehending eleven species (6,074 specimens), having a lower diversity when compared with the data by Gettinger et al. (2005) conducted in the Amazonian biome that presented five genera and twenty-one species with a significantly smaller number of specimens (1,014), however, when we compared it to another study conducted in the same biome as ours (Nieri-Bastos et al. 2004), the diversity is similar, reporting four genera and ten species distributed among 729 specimens collected. The biome difference and the abundance of preferential hosts can explain this event.

The genus *Ornithonyssus* was the only taxon belonging to the family Macronyssidae. The species included in this genus have hematophagous habits. Fonseca (1948) described eleven species in Brazil, with some species already synonymized over the following years. Nowadays, 5 species are valid and present in the Brazilian territory according to the checklist proposed by Bassini-Silva et al. (2020). In our study, we observed a host preference regarding this mite (*Ornithonyssuss* sp.). Mites were collected from three rodent genera and no specimens were retrieved from marsupials., Nieri-Bastos et al. (2011) reported of the same genus from eleven hosts in three Brazilian states. Sponchiado et al. (2015) studied the Cerrado biome and recorded a species of *Ornithonyssus* in the marsupial *Gracilinanus agilis* (Burmeister, 1854). Coinciding with Bassini-Silva et al. (2020), we recorded this genus in a preserved area inside the Atlantic Rainforest.

Many authors have reported the presence of *Rickettsia* DNA in mites collected from wild and synanthropic hosts belonging to the families reported in this study: Trombiculidae (Choi et al. 2007; Jacinavicius et al. 2019; Ponnusamy et al. 2022), Laelapidae (Miťková et al. 2015; Kuo et al. 2020) and Macronyssidae (Reeves et al. 2007). On the other hand, there was no amplification for the bacteria *Rickettsia* in our study. One hypothesis for this result is that the rickettsiae circulating in the collected area are not able to generate infection in the sampled mite species and can also be explained by the low amount of target DNA of this bacteria, a common fact when it comes to its main vector (Serpa et al. 2021).

## Conclusion

This study contributes to a better understanding of the relationships between ectoparasitic mites and small terrestrial mammals in the Vale do Ribeira region, inside a conservational area in the Atlantic Rainforest biome. We report new records of locality, host-parasite interactions, and the ectoparasitic fauna inside the area that had not been previously studied. This study’s highlight the importance of natural areas, their inner balance, and the harmful actions that urbanization may bring. Further research on other mites-associated pathogens is essential for epidemiological public health.

## Data Availability

The data supporting the findings of this study are available within the article. All the material used is housed at the Acarological Collection of Butantan Institute (IBSP) and is publicly available for examination.
